# The Predictive Role of Community of Inquiry in Shaping Students' Creative Self‐Concept: A Cross‐Sectional Study

**DOI:** 10.1002/nop2.70459

**Published:** 2026-02-12

**Authors:** Shaherah Yousef Andargeery, Norah Mohammed Alotaibi, Ghalia Mohammed Almadi, Layan Ali Alhazmi, Lama Fahad Alwehaibi, Razan Faisal Alkathiri, Sadeem Saleh Albalawi, Hatoon Hashan Alqahtani, Rana Mohammed Alsubaie, Murad H. Taani, Salwa Mohammad Maghrabi

**Affiliations:** ^1^ Department of Nursing Management and Education, College of Nursing Princess Nourah bint Abdulrahman University Riyadh Saudi Arabia; ^2^ College of Nursing Princess Nourah Bint Abdulrahman University Riyadh Saudi Arabia; ^3^ College of Health Professions & Sciences, School of Nursing University of Wisconsin‐Milwaukee Milwaukee Wisconsin USA; ^4^ Department of Clinical Nursing Practices, Faculty of Nursing Umm Al‐Qura University Makkah Saudi Arabia

**Keywords:** cognitive presence, community of inquiry, creative self‐concept, nursing education, social presence, teaching presence

## Abstract

**Background:**

Creative self‐concept is essential for nursing students as it supports innovation, adaptability and problem‐solving in clinical practice. The community of inquiry framework offers a model for promoting reflective and meaningful learning. This study explored the role of community of inquiry and demographics as predictors of creative self‐concept among Saudi nursing students.

**Methods:**

A cross‐sectional, correlational design was used with 394 nursing students from a public university in Riyadh, selected through stratified random sampling. Data was collected using validated self‐report instruments: the community of inquiry Survey and Short Scale of Creative Self.

**Results:**

Students reported moderate levels across community of inquiry subscales and moderately high Creative self‐concept. It was positively correlated with all community of inquiry dimensions, particularly the Resolution subscale. Regression analysis showed age, year of study, Design and Organisation and Resolution significantly predicted creative self‐concept (Adjusted *R* ^2^ = 0.182, *p* < 0.001).

**Conclusion:**

Findings suggest that student creativity‐related self‐beliefs are more strongly associated with instructional design and organisation, and with learning experiences that emphasise application and resolution than with general engagement alone. However, the modest proportion of explained variance indicates that creative self‐concept is likely shaped by additional personal and contextual factors beyond CoI, highlighting the need for curricula that cultivate creative confidence and for longitudinal research to clarify causal pathways.

**Public Contributions:**

Undergraduate nursing students indirectly contributed by providing feedback during data collection through their responses to the survey instruments.

## 1 Background

Creativity has emerged as a critical competency for nursing professionals in response to the growing complexity of healthcare systems, rapid technological advancements and increasing demands for patient‐centred and innovative care (Thabane et al. 2025; Cheraghi et al. 2021; Notarnicola et al. 2025). Contemporary nursing practice requires practitioners who can adapt to uncertainty, integrate diverse sources of information and generate context‐sensitive solutions to clinical problems (Göras et al. 2020; Lenssen et al. 2025). Consequently, nursing education has increasingly emphasised the development of higher order cognitive and psychological capacities that extend beyond technical proficiency and academic performance alone.

A key psychological construct underpinning creativity is creative self‐concept, that is defined as individuals' beliefs about their creative potential and identity as creative thinkers (Karwowski and Barbot 2016). Creative self‐concept encompasses both creative self‐efficacy, which is defined as the belief in one's creative capabilities, and creative personal identity, which refers to the extent to which creativity is central to one's self‐image (Karwowski 2016). These self‐beliefs develop through social, cognitive and reflective processes and are shaped by both individual and contextual factors (Karwowski 2016). Creative self‐concept plays a foundational role in creative behaviour by influencing individuals' motivation to engage in novel tasks, persist through uncertainty and apply creative thinking in real‐world contexts (Urban and Urban 2025; Anderson and Haney 2021). Empirical evidence indicates that students with stronger creative self‐concept demonstrate higher engagement, greater intrinsic motivation and a stronger willingness to explore innovative approaches in both learning and professional practice (Álvarez‐Huerta et al. 2025; Li et al. 2025).

In health professions education, creative self‐concept is particularly prominent, as students are expected to integrate theoretical knowledge, clinical reasoning and adaptive decision‐making within complex and dynamic care environments (Liu et al. 2020).

Despite this relevance, creativity in nursing education has traditionally been examined through skills‐based or performance‐oriented perspectives, such as creative problem‐solving or innovation training, with comparatively limited attention to students' creative self‐beliefs as a developmental learning outcome (Geok and Yee 2024; Liu 2021; Ma et al. 2018; Yang et al. 2023). Notably, nursing education research has focused more extensively on professional self‐concept than on creative self‐concept. Systematic reviews suggest that nursing students' professional self‐concept is generally moderate and shaped by a combination of internal traits and external educational environments (Xu et al. 2022). Similarly, pedagogical approaches characterised by strong teaching presence, supportive learning environments and growth‐oriented feedback have been shown to foster self‐confidence and professional values aligned with nursing practice (De Gagne et al. 2021). Although creative self‐concept is rarely measured directly in this body of work, these findings suggest that professional identity development may provide an important foundation for the emergence of creative self‐beliefs. Understanding how educational environments shape creative self‐concept is therefore essential for designing curricula that not only teach creative skills but also cultivate the confidence and identity required to apply creativity effectively in nursing practice.

## 2 Introduction

### 2.1 Community of Inquiry as a Framework for Creative Development

The community of inquiry (CoI) framework provides a well‐established theoretical model for understanding learning as a socially mediated and cognitively driven process (Swan 2019). The framework conceptualises effective learning environments as the dynamic interaction of three core presences: teaching presence, social presence and cognitive presence (Garrison et al. 2010). Teaching presence encompasses course design, organisation, facilitation and direct instruction (Anderson et al. 2019; Fiock 2020; Moore and Miller 2022). While social presence refers to learners' ability to project themselves socially and emotionally. Cognitive presence reflects the extent to which learners engage in inquiry processes that culminate in meaning construction and problem resolution (Anderson et al. 2019; Fiock 2020; Moore and Miller 2022).

Extensive empirical research has demonstrated that CoI presences are associated with positive educational outcomes, including student satisfaction, engagement, perceived learning and critical thinking (Martin et al. 2022; Alshammari and Alrehaili 2025; Ay and Dağhan 2023). More recent studies have extended the CoI framework beyond academic outcomes to include motivational, emotional and self‐regulatory processes, highlighting its relevance for understanding psychological dimensions of learning (Burbage et al. 2023; Kilis and Yıldırım 2018; Na et al. 2024; Yang et al. 2024; Zhang et al. 2023). Within nursing and health professions education, students commonly report moderate to high perceived levels of teaching, social and cognitive presence in online and blended learning contexts (Aslan and Turgut 2021; Martin et al. 2022); however, these perceptions vary depending on course design, instructional organisation and broader contextual disruptions such as those associated with the COVID‐19 pandemic. Importantly, although CoI has been widely applied to explain engagement and academic outcomes, there remains limited direct evidence linking CoI presences to creativity‐related self‐beliefs in nursing students.

### 2.2 Association between Community of Inquiry and Creative Self‐Concept

Theoretically, the CoI framework aligns closely with conditions known to support creativity and the development of creative self‐beliefs. Teaching presence, particularly through effective design and organisation, fosters clarity, autonomy and psychological safety, which are the key antecedents of creative confidence (Anderson et al. 2019; Han et al. 2022; Sajnani et al. 2020). Empirical studies in health professions education further indicate that teaching presence positively predicts learning self‐efficacy; that is a construct conceptually proximate to creative self‐concept (Burbage et al. 2023). Despite these findings, no prior study has directly examined the relationship between teaching presence subscales and creative self‐concept among nursing students.

Social presence may also contribute to creative self‐concept by enabling open communication, emotional expression and peer validation, which are conditions that support creative risk‐taking and self‐expression (Tan et al. 2022; Tang and Hew 2020). In online and blended learning contexts, social presence has demonstrated strong associations with self‐regulation and co‐regulation (shared metacognition), which reinforce learners' confidence and agency (Sadaf et al. 2022; Na et al. 2024). While social presence may not directly generate creative outcomes, it may provide a psychologically safe environment in which creative self‐beliefs can develop. Nevertheless, empirical evidence directly linking social presence to creative self‐concept in nursing education remains scarce.

Cognitive presence, particularly the higher‐order phases of integration and resolution, reflects reflective thinking, synthesis and application of knowledge, which are processes that are central to both creative thinking and creative self‐concept (Sadaf et al. 2021; Moore and Miller 2022; Pinkow 2022). Structural modelling studies have shown that cognitive presence is closely linked to self‐efficacy and metacognitive regulation and is positively predicted by teaching and social presence (Jia et al. 2022; Zhou 2022). Given the conceptual alignment between creative self‐concept and confidence in applying novel ideas to solve problems, cognitive presence may be a particularly salient predictor of creative self‐beliefs in nursing students. However, this relationship has not yet been empirically tested within the CoI framework in nursing education.

Beyond instructional and contextual influences, demographic and developmental factors may also shape creative self‐concept. Developmental research indicates that creative self‐efficacy and creative personal identity increase from late adolescence into early adulthood (Karwowski 2016), with age‐related growth in creative thinking observed during the transition from secondary to higher education (Ulger 2023). Academic achievement has similarly been linked to creativity‐related self‐beliefs, with higher creative self‐efficacy reported among higher‐achieving students and those with stronger academic self‐concept (Roth et al. 2022; Ellala et al. 2023). Additionally, creative self‐concept may vary across stages of academic progression, with first‐year students' creative self‐beliefs more closely tied to reflective and higher‐order learning, and later years influenced by assessment demands and curricular structure (Álvarez‐Huerta et al. 2021; Mangion and Riebel 2023). Despite these findings, demographic predictors have rarely been examined alongside CoI presences in nursing education.

### 2.3 Research Gap and Significance

Despite growing interest in creativity and inquiry‐based learning, several critical gaps remain in the literature. First, although nursing education research indicates that students typically report moderate to high teaching and cognitive presence, and moderate social presence, there is little direct quantitative evidence examining creative self‐concept among nursing students. Much of the existing evidence is indirect and inferred from related constructs such as professional self‐concept and reflective, presence‐rich pedagogies (Xu et al. 2022; De Gagne et al. 2021; Froneman et al. 2023). Second, CoI research has historically emphasised academic and cognitive outcomes, with limited attention to creativity‐related psychological constructs, particularly creative self‐concept. Recent advances in creativity research highlight the importance of distinguishing creative self‐concept from creative performance and emphasise the use of validated, theoretically grounded measurement tools (Karwowski et al. 2019; Haase et al. 2018). However, few studies have integrated these contemporary conceptualisations into nursing education research using established learning frameworks such as CoI, and evidence from Middle Eastern nursing education contexts, including Saudi Arabia, remains limited.

Third, while no study to date has measured creative self‐concept alongside CoI subscales, adjacent research demonstrates robust positive associations between CoI presences and self‐efficacy, metacognition and self‐regulated learning, which are constructs that are conceptually close to creative self‐concept (Burbage et al. 2023; Jia et al. 2022; Zhou 2022; Yu and Li 2022). These findings suggest plausible links between CoI presences and creative self‐concept, yet such associations remain empirically untested and should be framed as a clear research gap rather than assumed from related variables. In summary, although nursing students commonly report moderate to high levels of CoI presences and moderate professional self‐concept, direct quantitative evidence on creative self‐concept remains scarce. Addressing these gaps, the present study investigates perceived levels of community of inquiry presences and examines the predictive role of CoI subscales and demographic characteristics in shaping creative self‐concept among pre‐registration nursing students in Saudi Arabia.

### 2.4 Aim of the Study

This study is conducted to examine whether community of inquiry dimensions and selected demographic variables predict creative self‐concept among Saudi pre‐registration undergraduate nursing students.

### 2.5 Research Questions

1. What are the perceived levels of teaching presence, social presence, cognitive presence and creative self‐concept among nursing students?
2. Do creative self‐concept scores differ by age group, GPA category and year of study?
3. What are the associations between community of inquiry subscales and creative self‐concept?
4. Which community of inquiry subscales (controlling for demographics) significantly predict creative self‐concept?

### 2.6 Hypothesis Development

*Nursing students will report measurable levels of teaching presence, social presence, cognitive presence, and creative self‐concept*.

*Creative self‐concept scores will significantly differ according to students' age group, GPA category, and year of study*.

*Teaching presence, social presence, and cognitive presence will be significantly associated with creative self‐concept among nursing students*.

*Teaching presence, social presence, and cognitive presence will significantly predict creative self‐concept after controlling for demographic variables (age, GPA, and year of study)*.

By empirically testing these hypotheses, the present study contributes to the literature by extending the application of the CoI framework to creativity‐related psychological outcomes and providing evidence to inform pedagogical strategies aimed at fostering creative confidence in nursing education.

## 3 Methodology

### 3.1 Design

This study employed a quantitative, non‐experimental, cross‐sectional correlational design to examine the predictive role of CoI presences and selected demographic variables on creative self‐concept among pre‐registration nursing students. A quantitative approach was deemed appropriate because it allows for objective measurement of psychological constructs, statistical examination of relationships among variables and identification of predictors within a large sample (Cathala and Moorley 2018). This design is particularly suitable when the goal is to test theoretically grounded hypotheses and quantify the relative contribution of multiple independent variables to a psychological outcome.

### 3.2 Setting and Sampling

This study was conducted at a selected public university in Riyadh, Saudi Arabia and targeted pre‐registration undergraduate Bachelor of Science (BSc) nursing students enrolled in the College of Nursing. Inclusion criteria encompassed currently enrolled undergraduate nursing students from first to fourth year, while students in internship or graduate programmes were excluded from participation.

The total population consisted of 1505 students across the first to fourth academic years. A sample size was determined using G*Power and Epi Info software, yielding a required minimum of 373 participants to ensure a 95% confidence level and a 5% margin of error. The final calculated minimum sample size for stratified sampling was 307 participants.

To ensure representation across academic years, we used stratified recruitment with proportional targets for each year level. Within each stratum, participants were recruited through purposive/convenience enrollment until the target number for that stratum was achieved. This approach is consistent with applied health education research when the aim is to achieve adequate representation across key subgroups under real‐world access constraints (Shorten and Moorley 2014). A priori sample size estimation indicated that the minimum required sample was met, and the final sample included 394 participants. The distribution of the sample across academic years, calculated using Slovin's formula, was as follows:

First year = 307/1505 × 557 = 113.62≈114 students.

Second year = 307/1505 × 476 = 97.09≈97 students.

Third year = 307/1505 × 317 = 64.66≈65 students.

Fourth year = 307/1505 × 155 = 31.61≈32 students.

This approach ensured balanced representation of the student body while accommodating practical constraints related to participant availability.

### 3.3 Variables of the Study

The dependent variable was creative self‐concept, conceptualised as students' beliefs about their creative abilities and creative identity. The independent variables consisted of: (1) Demographic variables, including age, cumulative grade point average (GPA) and year of study; (2) Community of inquiry presences, including teaching presence, social presence and cognitive presence.

### 3.4 Measurement

#### 3.4.1 Demographic Variables (Independent Variables)

A demographic questionnaire was used to collect participants' age, cumulative GPA and year of study. These variables were included based on prior evidence suggesting their potential influence on creativity‐related self‐beliefs and educational engagement.

#### 3.4.2 Community of Inquiry Presences (Independent Variables)

The community of inquiry survey, originally developed by Garrison et al. (2000) and validated by Arbaugh et al. (2008), was used to measure students' perceptions of teaching, social and cognitive presence. The CoI has three main dimensions. Teaching presence was assessed through three subscales: design and organisation, facilitation and direct instruction. Social presence was assessed through: affective expression, open communication and group cohesion. Cognitive presence was assessed through: triggering event, exploration, integration and resolution.

Participants responded using a 5‐point Likert scale ranging from 1 (strongly disagree) to 5 (strongly agree). Subscale scores were calculated by averaging item responses, with higher scores indicating stronger perceived presence. The instrument demonstrated excellent internal consistency in the current study (overall Cronbach's *α* = 0.963) (Table 2).

### 3.5 Creative Self‐Concept (Dependent Variable)

Creative self‐concept was measured using the Short Scale of Creative Self, developed and validated by Lamb et al. (2025). This instrument consists of 11 items assessing creative self‐efficacy and creative personal identity using a 5‐point Likert scale ranging from 1 (definitely not) to 5 (definitely yes). An overall creative self‐concept score was derived by averaging the responses to all 11 items. Higher scores reflect a stronger identification with creativity and greater confidence in one's creative abilities. The scale demonstrated excellent reliability in the current sample (Cronbach's *α* = 0.942) (Table 2).

### 3.6 Pilot Testing

Prior to full‐scale data collection, a pilot test was conducted to evaluate the clarity, feasibility and technical functionality of the survey instrument within the study context. The pilot involved a small group of 20 pre‐registration undergraduate nursing students who met the study inclusion criteria but were not included in the final sample.

The primary objectives of the pilot test were to assess: (a) clarity and comprehensibility of survey items and instructions; (b) appropriateness of item wording for the local educational and cultural context; (c) estimated time required to complete the questionnaire; and (d) functionality and accessibility of the online survey platform. Feedback from pilot participants indicated that the items were clear, relevant and easy to understand, and no substantive ambiguities or comprehension difficulties were reported. The average completion time ranged between 7 and 10 min, which was deemed acceptable for student participation, minimising the risk of response burden. Based on pilot feedback, minor formatting and layout adjustments were made to enhance readability and navigation within the online survey. No changes were made to the content, structure or scoring of the standardised instruments as they demonstrated strong conceptual clarity and established psychometric validity. Data obtained from the pilot phase was not included in the final analysis.

### 3.7 Ethical considerations

Ethical approval for this study was obtained from the Institutional Review Board (IRB) of an academic institution in Riyadh, Saudi Arabia (Log Number: 24‐0769). Informed written consent was obtained from all participants prior to data collection. Participants were assured of the confidentiality and anonymity of their responses, and all data were handled in accordance with ethical research standards. Participation was entirely voluntary, and students were informed of their right to withdraw from the study at any time without penalty. Ethical safeguards were maintained throughout all phases of the study before, during and after data collection, to ensure the protection of participants' rights and well‐being.

### 3.8 Data collection

Data collection was conducted following ethical approval from the IRB of the selected academic institution and administrative clearance from the Dean of Student Affairs. The survey instrument was digitised using Microsoft Forms and distributed to participants via official university email accounts and WhatsApp, facilitated by the Student Affairs Office. The questionnaire required approximately 7 to 10 min to be completed. Data was collected over a period extending from 26 June 2024, to 11 January 2025.

### 3.9 Data Collection Procedure

Following Institutional Review Board approval, data was collected using an online survey distributed via official university communication channels. Participation was voluntary, and informed consent was obtained electronically prior to survey completion. The survey required approximately 7–10 min to complete and was administered during the regular academic semester to capture students' perceptions within an authentic learning context.

### 3.10 Data Analysis

Data were analysed using IBM SPSS Statistics version (28). Prior to hypothesis testing, data were screened for completeness, outliers and distributional assumptions. Missing data were assessed, and analyses were conducted using complete cases where appropriate. For inferential analyses, assumptions for parametric tests, including approximate normality and homogeneity of variance, were examined and met. Independent samples *t*‐tests and one‐way analyses of variance (ANOVAs) were performed to examine group differences in creative self‐concept based on demographic variables (age, GPA and year of study). Pearson product–moment correlation coefficients were used to assess the relationships among the subscales of the community of inquiry framework and creative self‐concept. For multiple linear regression analyses, assumptions of normality, linearity, homoscedasticity and multicollinearity were evaluated, and no violations were identified. Multiple linear regression was then performed to identify which community of inquiry subscales were significantly associated with students' creative self‐concept. Statistical significance was set at *p* < 0.05 for all analyses.

## 4 Results

### 4.1 Participants' Demographic Characteristics

A total of 394 participants were included in the study. As shown in Table 1, the ages of the participants range between 18 and 23, and the distribution showed that 49.2% (*n* = 194) of the participants were between 18 and 19 years old, while 50.8% (*n* = 200) were aged 20–23 years, indicating a nearly equal distribution across the two age groups. Regarding academic performance, 10.7% (*n* = 42) of the students had a GPA of ≥ 3.4, 65.0% (*n* = 256) had a GPA ranging from 3.5 to 4.4, and 24.4% (*n* = 96) had a GPA between 4.4 and 5. This suggests that the majority of participants were performing at a mid‐range GPA level.

**TABLE 1:** Descriptive statistics of the participants' demographic profile (*N* = 394).

Sociodemographic profile	Frequency	Percent
Age		
18–19	194	49.2
20–23	200	50.8
GPA		
≤ 3.4	42	10.7
3.5 to 4.4	256	65
4.4 to 5	96	24.4
Year of study		
1st year	150	38.1
2nd year	106	26.9
3rd year	79	20.1
4th year	59	15.0

In terms of the year of study, 38.1% (*n* = 150) were first year students, 26.9% (*n* = 106) were in their second year, 20.1% (*n* = 79) were third year students and 15.0% (*n* = 59) were in their fourth year. These findings indicate a higher representation of students in the earlier years of their academic programme.

### 4.2 Participants' Perceived Levels Across Subscales of Community of Inquiry and Creative Self‐Concept Scale

Participants' perceptions across each subscale were interpreted by comparing mean scores to the theoretical midpoints of their respective ranges (Table 2). Scores above the midpoint indicate a high perceived level, scores near the midpoint reflect a moderate level, while scores below suggest a low perceived level.

**TABLE 2:** Reliability and participants' perceived levels across subscales of COI and CSC scale.

Scales/Subscales	Cronbach's alpha	Range	Mean	SD
Community of inquiry	0.963			
Teaching presence	0.918			
Design and organisation	0.841	4–20	12.680	2.147
Facilitation	0.906	6–30	18.421	3.362
Direct instruction	0.829	3–15	9.124	1.849
Social presence	0.914			
Affective expression	0.738	3–15	9.627	1.656
Open communication	0.808	3–15	9.477	1.656
Group cohesion	0.749	3–15	9.353	1.794
Cognitive presence	0.929			
Triggering event	0.813	3–15	9.300	1.724
Exploration	0.780	3–15	9.459	1.680
Integration	0.829	3–15	9.546	1.584
Resolution	0.826	3–15	9.388	1.753
Creative self‐concept	0.942	11–55	37.980	6.474

#### 4.2.1 Teaching Presence

The Design and Organisation subscale (range = 4–20) had a mean score of 12.68 (SD = 2.15), indicating a moderate perception of how clearly instructional materials and course structure were presented. The Facilitation subscale (range = 6–30) had a mean of 18.42 (SD = 3.36), suggesting that participants perceived the facilitation of learning activities to be moderate. The Direct Instruction subscale (range = 3–15) had a mean of 9.12 (SD = 1.85), indicating a moderate perception of the instructor's provision of direct content‐related input.

#### 4.2.2 Social Presence

All three subscales under social presence had a possible range of 3–15. Affective Expression (*M* = 9.63, SD = 1.66), Open Communication (*M* = 9.48, SD = 1.66) and Group Cohesion (*M* = 9.35, SD = 1.79) each scored at the midpoint. These results suggest participants experienced moderate levels of emotional expression, open interaction and a sense of connectedness within the learning community.

#### 4.2.3 Cognitive Presence

The cognitive presence subscales also used a 3–15 range. The mean scores for Triggering Event (*M* = 9.30, SD = 1.72), Exploration (*M* = 9.46, SD = 1.68), Integration (*M* = 9.55, SD = 1.58) and Resolution (*M* = 9.39, SD = 1.75) were all at the midpoint. This indicates that participants perceived a moderate level of cognitive engagement, from the initial identification of problems to reflection and problem‐solving.

#### 4.2.4 Creative Self‐Concept

For the creative self‐concept scale (range = 11–55), the mean score was 37.98 (SD = 6.47), suggesting that participants had a slightly high self‐perception of their creative abilities. In summary, participants reported moderate to high levels of agreement across all subscales, reflecting overall positive perceptions of teaching, social and cognitive presence, as well as their own creative self‐concept within the learning environment.

### 4.3 Mean Differences in Creative Self‐Concept Based on Demographic Variables

An independent samples *t*‐test and one‐way ANOVA were conducted to examine differences in participants' creative self‐concept based on age, GPA and year of study (Table 3). The results revealed no statistically significant difference in creative self‐concept scores based on age, *t*(392) = 1.011, *p* = 0.313, indicating that participants' perceptions of their creative self‐concept did not significantly vary between different age groups. Similarly, a one‐way ANOVA showed no significant difference in creative self‐concept scores based on GPA, *F*(2, 391) = 0.813, *p* = 0.736, suggesting that academic performance levels were not associated with differing levels of creative self‐concept. These findings suggest that demographic variables such as age and GPA do not appear to significantly influence students' perceptions of their creative self‐concept.

**TABLE 3:** Mean differences in CSC based on demographic variables.

Demographics	Test	Sig. (2‐tailed)
Age	*t* = 1.011	0.313
GPA	*F* = 0.813	0.542
Year of study	*F* = 4.709	0.003*

* Statistically significant at *p* ≤ 0.05.

In contrast, a one‐way ANOVA comparing creative self‐concept scores across academic year levels revealed a statistically significant difference, *F*(3, 390) = 4.709, *p* = 0.003. This result suggests that nursing students' perceptions of their creative self‐concept differ by year of study. To determine which groups differed significantly, post hoc comparisons were conducted using both Tukey's HSD and Games–Howell procedures (Table 4). Third‐year students reported significantly higher creative self‐concept scores than first‐year students (Tukey: *M* difference = 3.23, *p* = 0.002; Games–Howell: *p* = 0.003) and second‐year students (Tukey: *M* difference = 2.67, *p* = 0.026).

**TABLE 4:** Post hoc comparisons of creative self‐concept (CSC) scores across year levels.

Group comparison	Mean difference	Standard error	*p*e	95% CI (Lower)	95% CI (Upper)
First year vs. second year	0.56	0.81	0.901	−1.53	2.65
First year vs. third year	3.23	0.89	0.002*	0.94	5.22
First year vs. fourth year	1.43	0.98	0.462	−1.1	3.97
Second year vs. third year	2.67	0.95	0.026*	0.23	5.12
Second year vs. fourth year	0.87	1.04	0.834	−1.8	3.55
Third year vs. fourth year	−1.8	1.1	0.358	−4.63	1.03

* Statistically significant at *p* ≤ 0.05.

However, no statistically significant differences were found between first year and second year students (*p* = 0.901), third year and fourth year students (*p* > 0.05), first year and fourth year students (*p* = 0.462) and between second year and fourth year students (*p* = 0.834). These findings suggest that third year nursing students perceive themselves as significantly more creative compared to students in the first and second years, while fourth year students' creative self‐concept levels were not significantly different from any group.

### 4.4 Correlational Relationships Among the Subscales of the Community of Inquiry and Creative Self‐Concept

Pearson correlation coefficients were computed to examine the relationships among the subscales of community of inquiry, creative self‐concept and their subscales (Table 5). All subscales within the teaching, social and cognitive presence dimensions of the community of inquiry model were significantly and positively correlated with each other (*p* < 0.01). Specifically, strong correlations were observed between Facilitation and Direct Instruction (*r* = 0.783, *p* < 0.01), Open Communication and Group Cohesion (*r* = 0.763, *p* < 0.01) and Integration and Resolution (*r* = 0.755, *p* < 0.01).

**TABLE 5:** Correlational relationships among the subscales of the CoI and CSC.

Scale/Subscales	1	2	3	4	5	6	7	8	9	10	11
1. Design and organisation	1										
2. Facilitation	0.766**	1									
3. Direct instruction	0.675**	0.783**	1								
4. Affective expression	0.552**	0.573**	0.575**	1							
5. Open communication	0.522**	0.536**	0.529**	0.716**	1						
6. Group cohesion	0.512**	0.514**	0.509**	0.638**	0.763**	1					
7. Triggering event	0.579**	0.561**	0.587**	0.584**	0.560**	0.638**	1				
8. Exploration	0.549**	0.552**	0.598**	0.506**	0.531**	0.561**	0.712**	1			
9. Integration	0.625**	0.580**	0.610**	0.511**	0.521**	0.527**	0.655**	0.731**	1		
10. Resolution	0.604**	0.590**	0.574**	0.527**	0.533**	0.556**	0.671**	0.694**	0.755**	1	
11. Creative self‐concept	0.325**	0.246**	0.247**	0.284**	0.276**	0.284**	0.339**	0.304**	0.329**	0.392**	1

** Correlation is significant at the 0.01 level (2‐tailed).

Creative self‐concept was found to have small to moderate, yet statistically significant, positive correlations with all community of inquiry subscales (*p* < 0.01). The strongest correlation was observed with resolution (*r* = 0.392, *p* < 0.01), followed by Integration (*r* = 0.329, *p* < 0.01) and triggering event (*r* = 0.339, *p* < 0.01), suggesting that higher levels of cognitive engagement are associated with stronger perceptions of creative self‐concept.

Among the teaching presence components, Design and Organisation (*r* = 0.325, *p* < 0.01) showed the highest correlation with creative self‐concept, while Facilitation and Direct Instruction showed weaker correlations (*r* = 0.246 and *r* = 0.247, respectively; *p* < 0.01). Similarly, within SP, Affective Expression, Open Communication and Group Cohesion each showed significant but moderate correlations with creative self‐concept (ranging from 0.276 to 0.284, *p* < 0.01). Overall, these findings support a significant, positive association between the community of inquiry framework and creative self‐concept, particularly highlighting the influence of cognitive presence dimensions.

### 4.5 Multiple Regression Predicting Creative Self‐Concept from Community of Inquiry Subscales

A multiple linear regression analysis was conducted to examine the extent to which demographic variables and community of inquiry subscales predicted creative self‐concept among nursing students (Table 6). The overall regression model was statistically significant, *F*(13, 380) = 7.73, *p* < 0.001, indicating that the predictors reliably explained variance in creative self‐concept. The adjusted *R* ^2^ was 0.182, suggesting that approximately 18.2% of the variance in creative self‐concept was accounted for by the combination of demographic and community of inquiry ‐related predictors.

**TABLE 6:** Multiple regression predicting CSC from demographics and CoI subscales (*n* = 394).

Predictors	*b*	*β*	*t*	*p*	95% CI	
					LL	UL
Age	1.880	0.145	2.196	0.029*	0.197	3.564
GPA	0.618	0.055	1.167	0.244	−0.423	1.658
Year of study	−1.401	−0.234	−3.549	0.000*	−2.177	−0.625
Design and organisation	0.584	0.194	2.501	0.013*	0.125	1.043
Facilitation	−0.272	−0.141	−1.602	0.110	−0.605	0.062
Direct instruction	−0.087	−0.025	−0.309	0.757	−0.642	0.468
Affective expression	0.143	0.036	0.505	0.614	−0.413	0.698
Open communication	0.156	0.043	0.534	0.594	−0.417	0.729
Group cohesion	−0.074	−0.020	−0.254	0.799	−0.650	0.501
Triggering event	0.368	0.103	1.356	0.176	−0.166	0.902
Exploration	0.010	0.002	0.032	0.975	−0.580	0.599
Integration	0.048	0.012	0.143	0.886	−0.604	0.700
Resolution	0.973	0.263	3.381	0.001*	0.407	1.538
Adjusted *R* ^2^ = 0.182, *F* (13, 380) = 7.732, *p* < 0.001*						

* Statistically significant at *p* ≤ 0.05.

Among the demographic variables, age was a significant positive predictor of creative self‐concept (*b* = 1.88, *β* = 0.145, *t* = 2.20, *p* = 0.029), indicating that older students tended to report higher levels of creative self‐concept. Conversely, year of study was a significant negative predictor (*b* = −1.40, *β* = −0.234, *t* = −3.55, *p* < 0.001), suggesting that students in more advanced academic years reported lower creative self‐concept scores. However, GPA was not a statistically significant predictor (*p* = 0.244).

Among the community of inquiry subscales, the design and organisation subscale was positively associated with creative self‐concept (*b* = 0.584, *β* = 0.194, *t* = 2.50, *p* = 0.013), indicating that structured instructional design contributed significantly to students' creative self‐concept. Additionally, the resolution subscale was a strong positive predictor of creative self‐concept (*b* = 0.973, *β* = 0.263, *t* = 3.38, *p* = 0.001), reflecting that the more students have the ability to apply knowledge and reach conclusions or solutions to problems in a learning environment, the more they see themselves as creative individuals. No other community of inquiry subscales reached statistical significance in the model. These findings highlight the importance of age, academic progression, instructional design and cognitive resolution in shaping nursing students' perceptions of their creative capacities within the learning environment.

## 5 Discussion

This study examined the predictive role of CoI presences and demographic characteristics in shaping creative self‐concept among pre‐registration nursing students, specifically exploring relationships between perceived learning environment characteristics and students' creative self‐beliefs within an existing educational context. The results extend current understanding of creativity in nursing education by positioning creative self‐concept as a meaningful psychological outcome that can be explained, in part, through established learning environment frameworks such as CoI. While prior nursing education research has largely emphasised academic achievement, engagement or professional identity development, the present findings suggest that the psychological conditions fostered by inquiry‐based learning environments also contribute to how nursing students perceive their own creative abilities and identities.

One of the central theoretical contributions of the results lies in demonstrating that selected CoI presences, particularly teaching presence and cognitive presence, are significant predictors of creative self‐concept. This finding extends the CoI framework beyond its traditional focus on cognitive and academic outcomes to include creativity‐related self‐beliefs, thereby responding to recent calls to examine the psychological dimensions of learning more explicitly (Burbage et al. 2023; Yu and Li 2022).

Teaching presence, specifically the design and organisation component, emerged as a significant predictor of creative self‐concept. From a theoretical perspective, this finding supports the argument that structure and clarity function as enabling conditions for creativity rather than as constraints. Well‐designed learning environments may reduce cognitive ambiguity, enhance students' sense of autonomy and promote psychological safety, which are conditions that creativity theory identifies as essential for the development of creative confidence and identity. This aligns with broader educational research suggesting that creativity is most likely to flourish in environments where expectations are clear and learners feel supported to take intellectual risks within a coherent instructional framework.

Cognitive presence, particularly the resolution phase, demonstrated the strongest predictive relationship with creative self‐concept. Resolution represents learners' perceived ability to apply knowledge to solve problems and reach meaningful conclusions. Theoretically, this finding aligns with contemporary models of creative self‐concept, which emphasise that creative self‐beliefs are strengthened not merely through idea generation or exploration, but through successful application and implementation of ideas (Karwowski 2016; Karwowski et al. 2019). This suggests that creative self‐concept in nursing students may be reinforced when learners perceive themselves as capable of translating inquiry and reflection into effective clinical reasoning and problem‐solving. From a CoI perspective, this finding refines existing interpretations of cognitive presence by highlighting resolution as not only a cognitive outcome, but also a psychologically consequential phase that shapes learners' creative self‐beliefs.

In contrast, social presence did not emerge as a significant direct predictor of creative self‐concept in the regression model. This finding does not negate the importance of social presence but rather suggests a more nuanced theoretical role. Prior research indicates that social presence supports psychological safety, validation and collaborative engagement, which may influence creativity‐related outcomes indirectly through mechanisms such as self‐efficacy, metacognition or self‐regulated learning (Sadaf et al. 2022; Na et al. 2024). From this perspective, social presence may function as a contextual enabler that creates favourable conditions for creative self‐concept development, rather than as a direct determinant. This interpretation contributes to theory by suggesting that CoI presences may operate through distinct pathways depending on the psychological outcome under investigation.

### 5.1 Demographic Factors and Creative Self‐Concept

The findings also provide theoretical insights into the role of demographic and developmental variables in shaping creative self‐concept. Age emerged as a significant predictor, consistent with developmental research demonstrating increases in creative self‐efficacy and creative personal identity from late adolescence into early adulthood (Karwowski 2016). This supports the view that creative self‐concept is a developmental construct that evolves alongside cognitive maturation and educational experiences. Differences associated with year of study further underscore the dynamic nature of creative self‐concept within professional education. The observed patterns may reflect increasing academic and assessment pressures in later stages of nursing programmes, which creativity research suggests can suppress creative self‐beliefs when learning becomes more performance‐driven and less exploratory (Álvarez‐Huerta et al. 2021). This finding highlights the importance of sustaining inquiry‐oriented and reflective learning opportunities throughout the curriculum, rather than concentrating them in early programme stages.

The association between academic achievement (GPA) and creative self‐concept aligns with prior evidence linking higher creative self‐efficacy to stronger academic self‐beliefs and performance status (Roth et al. 2022; Ellala et al. 2023). From a theoretical standpoint, this suggests that academic success and creative self‐beliefs may be mutually reinforcing, particularly in demanding professional programmes such as nursing. However, the presence of creative self‐concept among lower‐achieving students in previous research also indicates that creativity‐related self‐beliefs should not be viewed as the exclusive domain of high achievers, but rather as a malleable psychological resource that can be supported through appropriate learning environments.

### 5.2 Implications

The study's findings offer important theoretical and applied insights into how learning environments support creativity‐related self‐beliefs in contemporary higher education.

#### 5.2.1 Theoretical Implications: Extending the Community of Inquiry Framework

The findings extend the community of inquiry framework by demonstrating its relevance beyond traditional cognitive and academic outcomes to include creative self‐concept as a psychological learning outcome. While CoI has been widely applied to explain engagement, satisfaction and critical thinking, this study provides empirical support for its role in shaping students' creative self‐beliefs, particularly through teaching presence (design and organisation) and cognitive presence (resolution).

The prominence of cognitive presence, specifically the resolution phase, aligns with contemporary educational research emphasising higher‐order thinking, metacognitive regulation and adaptive knowledge application. Resolution reflects learners' ability to integrate ideas, apply knowledge and reach reasoned conclusions, which are processes that are conceptually aligned with creative self‐efficacy and creative identity. This finding resonates with recent studies highlighting the importance of self‐regulated learning, cognitive flexibility and metacognitive awareness in shaping students' confidence, attitudes and emotional responses in digitally mediated learning environments, including those involving generative artificial intelligence (Bai and Gu 2025; Moore and Miller 2022; Sadaf et al. 2021).

Recent work exploring students' anxiety and attitudes toward generative AI has shown that cognitive flexibility, self‐regulation skills and metacognitive awareness play a central role in shaping how learners perceive and engage with complex, emerging technologies (Ren et al. 2025; Yılmaz et al. 2025). These constructs function as psychological resources that enable learners to navigate uncertainty, adapt to novel tasks and maintain confidence, which are mechanisms that closely parallel the role of creative self‐concept identified in the present study. Viewed through this lens, creative self‐concept may represent a complementary psychological capacity that supports adaptive learning in technologically complex and rapidly evolving educational contexts.

#### 5.2.2 Teaching Presence, Pedagogical Design and Contemporary Models of Learning

The significant predictive role of design and organisation underscores the importance of intentional pedagogical design in fostering creative self‐beliefs. Well‐structured learning environments provide clarity, reduce cognitive overload and support learner autonomy, conditions that are increasingly recognised as essential in digitally intensive and AI‐augmented learning environments. This finding aligns with broader pedagogical scholarship calling for adequate and future‐oriented models of pedagogy, particularly those that balance structure with flexibility, guidance with autonomy and technological affordances with human‐centred design. In this context, teaching presence operates not merely as instructional delivery, but as a regulatory scaffold that supports students' self‐regulation, creative confidence and willingness to engage in complex problem‐solving.

Importantly, while social presence did not emerge as a significant predictor in the regression model, its consistent positive correlations with creative self‐concept suggest a supportive but indirect role. This mirrors findings in studies of self‐regulated learning and AI‐related attitudes, where social and emotional dimensions often function as enabling conditions rather than direct predictors (Dahri et al. 2024; Mirriahi et al. 2025; Wang et al. 2025). Social presence may therefore contribute to psychological safety and emotional regulation, which are prerequisites for creative risk‐taking and cognitive flexibility.

#### 5.2.3 Broader Educational Implications Beyond Nursing

Although conducted within nursing education, the findings have broader relevance across disciplines, particularly in programmes characterised by online or blended learning, inquiry‐based pedagogies and increasing integration of digital and AI‐enabled tools. Creative self‐concept, like self‐regulation and cognitive flexibility, is not discipline‐specific. Rather, it represents a transferable psychological capacity that supports learners' adaptability, confidence and engagement across educational contexts (Álvarez‐Huerta et al. 2021; Anderson and Haney 2021). As higher education increasingly grapples with generative AI, rapid technological change and uncertain professional futures, fostering students' creative self‐beliefs becomes an essential pedagogical goal.

From this perspective, the CoI framework offers a robust, integrative model for designing learning environments that promote not only academic success but also adaptive psychological outcomes, including creativity, confidence and metacognitive control. The present findings suggest that strengthening cognitively demanding, resolution‐oriented learning activities, alongside structured instructional design, may help students across disciplines develop the confidence needed to engage creatively with complex and emerging challenges.

### 5.3 Limitations

This study has several limitations. The cross‐sectional, correlational design limits the ability to establish causal relationships between CoI presences and creative self‐concept. Longitudinal and experimental designs are needed to assess how changes in learning environments over time influence the development of creative self‐concept and to strengthen internal validity. Furthermore, the exclusive reliance on self‐report measures introduces potential response biases, including social desirability and recall bias. Although the instruments demonstrated strong psychometric properties, participants' perceptions of CoI presences and creative self‐concept may not fully reflect actual instructional practices or creative behaviours. Future studies should incorporate mixed‐method or objective measures to strengthen construct validity. In addition, creative self‐concept was assessed using a general measure that does not capture domain‐specific creativity in nursing or clinical contexts. As a result, the findings reflect global creative self‐beliefs rather than nursing‐specific creative competence, highlighting the need for validated discipline‐specific creativity measures. The sample characteristics present additional limitations. The sample comprised pre‐registration nursing students from a single institution in Saudi Arabia, which may limit generalisability to other educational, institutional or cultural contexts. Accordingly, the findings offer analytical rather than statistical generalisability. Although basic demographic variables were included, other relevant factors such as prior educational experiences, instructional characteristics and institutional support were not examined and may have introduced unmeasured confounding effects.

The generalisability of the findings should be interpreted with caution. This study was conducted within a single institutional and cultural context, involving pre‐registration nursing students enrolled in an undergraduate program in Saudi Arabia. Educational structures, pedagogical practices, gender composition and cultural norms in this context may differ from those in other nursing programmes nationally and internationally. As a result, the findings may not be directly transferable to mixed‐gender cohorts, postgraduate nursing students or institutions with different curricular models or learning technologies. Nevertheless, because the community of inquiry framework and creative self‐concept are theoretically grounded and internationally validated constructs, the observed relationships may hold conceptual relevance beyond the immediate study context. The findings therefore offer analytical rather than statistical generalisability, providing insights that may inform hypothesis development, curriculum design and further empirical testing in comparable educational settings.

## 6 Conclusion

Students reported moderate to high levels of teaching and cognitive presence and moderate social presence, consistent with prior CoI studies on health professions education. Importantly, creative self‐concept was significantly associated with teaching presence, particularly course design and organisation and cognitive presence, especially the resolution phase, indicating that students' creative self‐beliefs are shaped not only by individual characteristics but also by structured learning environments and enacted inquiry processes. By directly measuring creative self‐concept, this study extends the CoI framework beyond engagement and cognitive outcomes to creativity‐related psychological constructs and highlights the importance of successful knowledge application and problem‐solving in strengthening creative self‐beliefs. Although social presence did not emerge as a direct predictor, it may function as an indirect contextual facilitator, while associations with age, academic achievement and year of study suggest that creative self‐concept develops over time and is responsive to curricular demands. Overall, the findings provide empirical support for the relevance of teaching and cognitive presence in fostering creative self‐concept in nursing education and offer transferable theoretical insights for designing inquiry‐based learning environments in nursing and health professions education.

## Author Contributions

N.M.A., G.M.A., L.A.A., L.F.A., R.F.A., S.S.A., H.H.A., and R.M.A.: conceptualisation. S.Y.A., N.M.A., G.M.A., L.A.A., L.F.A., R.F.A., S.S.A., H.H.A., and R.M.A.: methodology. S.Y.A. and M.H.T.: software. S.Y.A. and M.H.T.: validation. S.Y.A. and M.H.T.: formal analysis. S.Y.A., M.H.T. and S.M.M.: investigation. S.Y.A., N.M.A., G.M.A., L.A.A., L.F.A., R.F.A., S.S.A., H.H.A. and R.M.A.: resources. S.Y.A. and M.H.T.: data curation. S.Y.A., N.M.A., G.M.A., L.A.A., L.F.A., R.F.A., S.S.A., H.H.A., R.M.A., M.H.T. and S.M.M.: writing: original draft preparation. S.Y.A., N.M.A., G.M.A., L.A.A., L.F.A., R.F.A., S.S.A., H.H.A., R.M.A, M.H.T. and S.M.M.: writing: review and editing. S.Y.A. and M.H.T.: visualisation. S.Y.A. and N.M.A.: supervision. N.M.A., G.M.A., L.A.A., L.F.A., R.F.A., S.S.A., H.H.A., and R.M.A.: project administration. S.Y.A.: funding acquisition. All authors have read and agreed to the published version of the manuscript.

## Funding

The authors extend their appreciation to Princess Nourah bint Abdulrahman University Researchers Supporting Project number (PNURSP2026R447), Princess Nourah bint Abdulrahman University, Riyadh, Saudi Arabia.

## Ethics Statement

IRB of a selected academic institution located in Saudi Arabia approved the study (Log Number: 24–0769).

## Consent

Informed consent was obtained from the study participants. There is no identifiable information on the participants reported within the manuscript.

## Conflicts of Interest

The authors declare no conflicts of interest.

## Data Availability

The datasets generated and/or analysed during the current study are not publicly available due to data privacy but are available from the corresponding author upon reasonable request.
